# Angiolymphoid hyperplasia with eosinophilia with clinical pictures of keratoacanthoma: A rare case report

**DOI:** 10.1002/ccr3.1949

**Published:** 2018-12-04

**Authors:** Calvin Santosa, Made Wardhana, Herman Saputra

**Affiliations:** ^1^ St. Carolus Hospital Jakarta Indonesia; ^2^ Sanglah General Public Hospital Denpasar Indonesia

**Keywords:** angiolymphoid hyperplasia with eosinophilia, dermatopathology, dermatosurgery, dermoscopy

## Abstract

Angiolymphoid Hyperplasia with Eosinophilia (ALHE) is a benign tumor of the skin which may mimic other benign or malignant lesion of the skin. Practitioners should consider this diagnosis as a differential when encounter such lesion as papule or nodule on the face and scalp area.

## 1 INTRODUCTION

Angiolymphoid hyperplasia with eosinophilia (ALHE) or used to be known as epitheloid hemangioma is a benign vascular disorder which clinically may mimic other diseases such as cylindroma, basalioma, or keratoacanthoma. This entity described in 1969 by Well and Whimster which at first is considered to be connected with Kimura Disease, not until later in 1980 accepted that both conditions have different clinical and histological pictures.^1^

Clinically ALHE present as solitary or multiple papules/nodules with dome shape and mostly occur in head and neck area. Clinically, it may mimic other diseases which makes diagnosis become difficult.^2^ Misdiagnosis of this entity is related to the high recurrence of the disease after initial treatment. Dermoscopy and histopathological examination are needed to diagnose this entity which gives out a better cure rate.^3^

## 2 CASE

A 55‐year‐old man presented with nodule on the nose since 7 months ago. The patient has a history of constant sun exposure due to his work as a farmer. History of bleeding when the patient rubs the lesion is mentioned. One year ago, he was diagnosed with keratoacanthoma and treated for the same lesion on the same area with electrocauterization.

**Figure 1:**
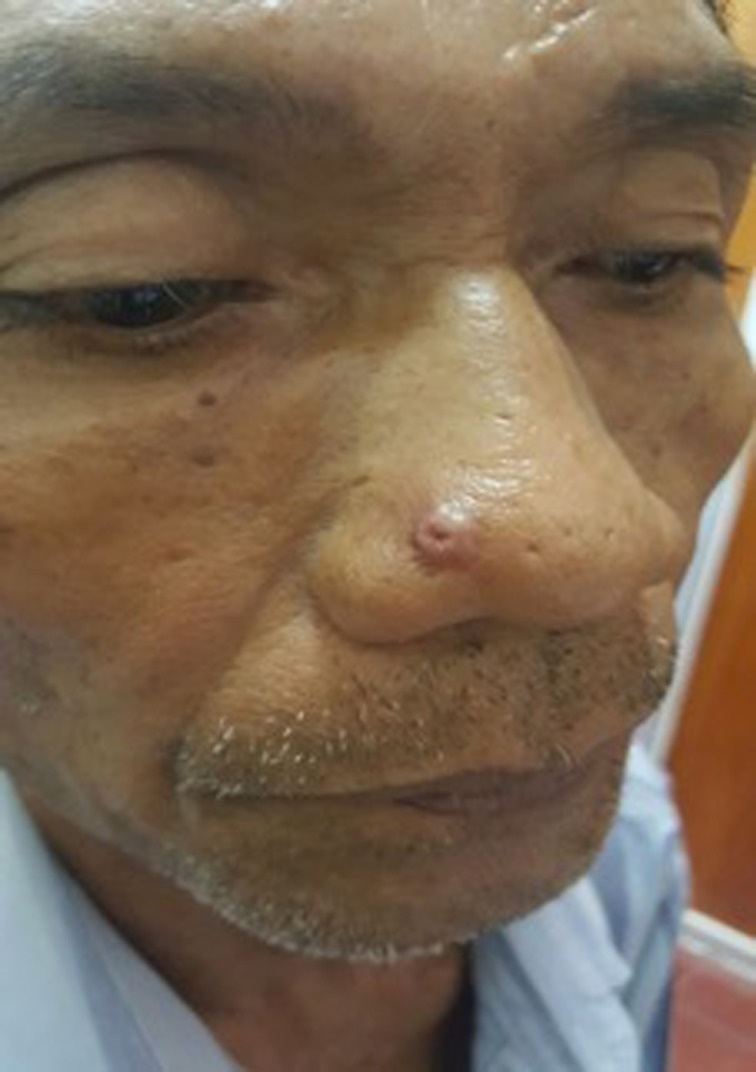
Clinical picture of solitary nodule with central ulceration on nasal area

**Figure 2:**
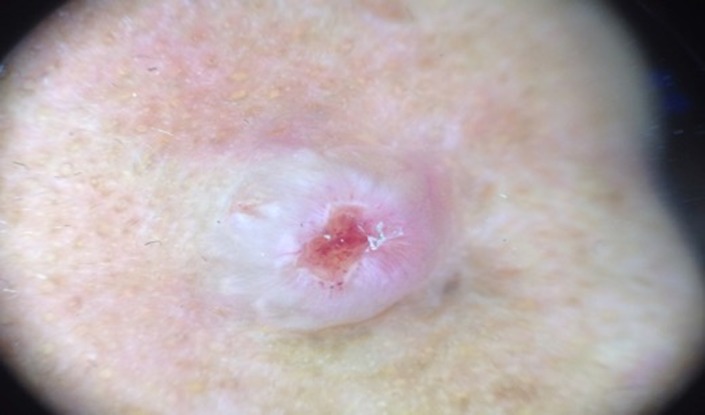
Dermoscopic picture which show multiple dotted vessels on the central ulcer, with white keratin mass on the adjacent area

On physical examination, there was a solitary nodule with 0.5 cm in diameter with solitary ulcer on top of it. (Figure 1) Dermoscopic examination shows keratin mass with pink background and ulcer on the central area. Vascular features such as dots and globular were also seen. The dermoscopic features were suitable for a keratoacanthoma (Figure 2).

**Figure 3:**
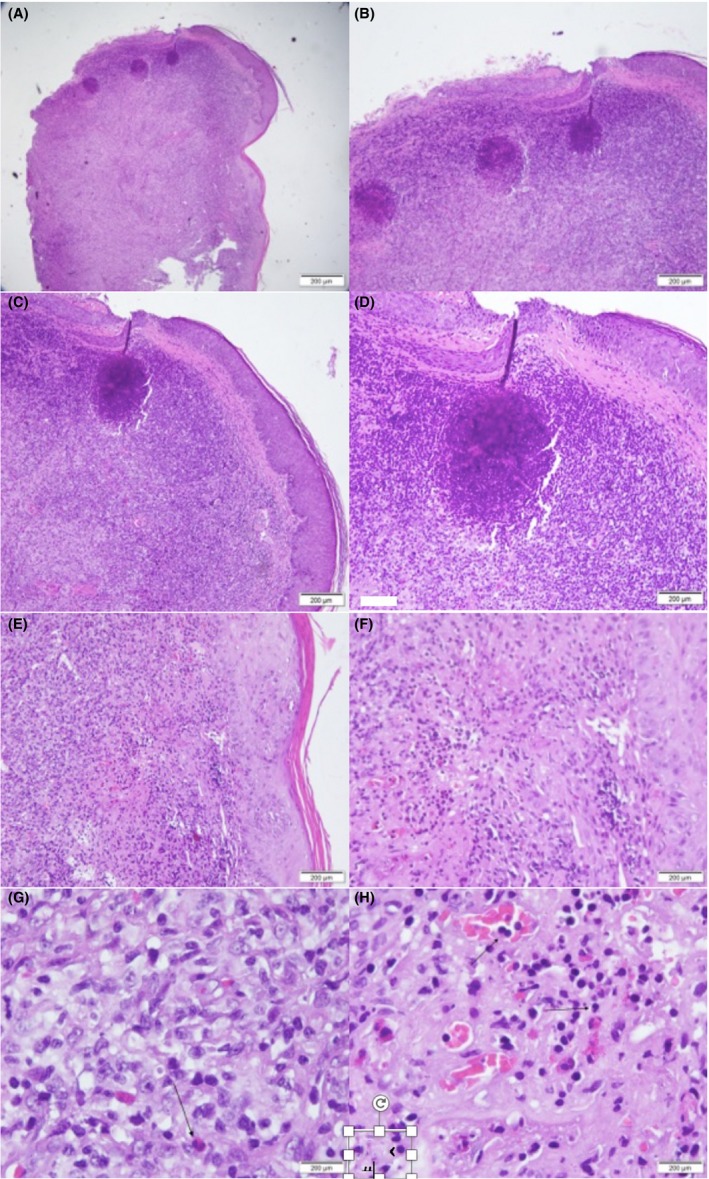
Histopathological examination (H&E stain 40x to 100x) showing dermal mass which consist of enlarged endothelial cells with infiltrate of eosinophils, consistent with diagnosis of Angiolymphoid Hyperplasia with Eosinophilia

From the histopathological examination shows a multiple mass with proliferation of capillary vessels, lymphoid cells and eosinophils infiltration which consistent with the diagnosis of angiolymphoid hyperplasia with eosinophilia (Figure 3A‐H) The patient was done an L‐plasty, and there is no sign of recurrence up to this day.

## 3 DISCUSSION

Angiolymphoid hyperplasia with eosinophilia (ALHE) epidemiologically tends to happen more in female compare to male. This condition has a high prevalence in the third to fifth decade of life. ALHE mostly occurs in the preauricular area (36%) followed by facial area (28,2%) and scalp (17,3%). Patient usually does not have any complain however itch which followed by bleeding and pain could be mentioned.^4^

The etiology and pathogenesis of ALHE is still unknown; however, researcher believed that this entity is formed due to vascular malformation. Some cases of ALHE have been connected into trauma history and Human T‐cell Lymphotropic virus (HTLV) or Human Herpesvirus 8 (HHV‐8) infection.^5^, ^6^, ^7^

Similar clinical appearance to other diseases has been the main challenge of diagnosing and treating this disease. There are some cases which shown ALHE with a clinical picture of cylindroma, pyogenic granuloma, and keratoacanthoma, which can be diagnosed only after a histopathological examination.^8^, ^9^, ^10^

From dermoscopic examination, we could find polymorphic vascular pattern which consist of dotted and irregular linear vascular pattern on top of pinkish area. Histopathological examination is the golden examination to diagnose this entity, where we will see vascular proliferation with enlarge endothelial cells and full of inflammatory response cells, mostly eosinophils. Different phase of the disease shows different histopathological pictures, in early phase we will see more vascular component, where in later stage there will be more inflammatory component around mature smaller endothelial cells.^11^

Treatment options are cryosurgery, pulse‐dye laser, topical tacrolimus, and surgery. Excisional surgery is the one with lower recurrence and higher cure rate compared to others. Imperfect excision from the operator may increase the recurrence of this disease.^12^

## CONFLICT OF INTEREST

None declared.

## AUTHOR CONTRIBUTION

CS: is the main author, diagnosed and examined the subject. MW: reviewed the final paper and done the excision of the lesion. HS: reviewed the histopathological sample of the biopsy of the lesion.
